# Clinical AI Beyond Development: A Scoping Review of Deployment-Related Robustness, Algorithmovigilance, and Lifecycle Oversight

**DOI:** 10.3390/healthcare14142052

**Published:** 2026-07-08

**Authors:** Rabie Adel El Arab, Mohammad Hussein Mustafa, Wesam Taher Almagharbeh, Mohammad Yahya Ayoub, Fatimah Alsanawi, Fulwa Almathen, Rawan Almosabeh, Magfrah Al Talaq

**Affiliations:** 1 Almoosa College of Health Sciences, Al Ahsa 36422, Saudi Arabia; 2 Dr. Sulaiman Alhabib Medical Group, Riyadh 71491, Saudi Arabia; 3 Medical and Surgical Nursing Department, Faculty of Nursing, University of Tabuk, Tabuk 71491, Saudi Arabia

**Keywords:** clinical artificial intelligence, post-development evaluation, post-deployment monitoring, algorithmovigilance, deployment-related robustness, lifecycle oversight, real-world evaluation

## Abstract

Background/Objectives: Clinical artificial intelligence (AI) is increasingly moving from proof-of-concept development into clinical evaluation, regulatory review, and routine care. This scoping review aimed to map and synthesise empirical evidence on clinical AI evaluation after model development, focusing on deployment-related robustness, post-development monitoring, and lifecycle oversight in practice. Methods: We conducted a scoping review in accordance with Joanna Briggs Institute guidance and reported findings using PRISMA-ScR. MEDLINE, Embase, Scopus, and Web of Science Core Collection were searched with no lower date restriction within each database’s available indexed coverage and with a common upper search date of 28 February 2026. Searches were supplemented by backward and forward citation tracking. Grey literature, preprint servers, and regulatory databases were not systematically searched because eligibility was restricted to full-text, peer-reviewed empirical studies and empirically grounded implementation or monitoring reports. Findings were synthesised using descriptive evidence mapping and inductive thematic synthesis. Results: Eighteen studies or empirically grounded reports were included. Evidence was organised into five strata: direct live or post-deployment monitoring studies; near-live bridge studies generating prospective outputs without guiding care; methodological monitoring and maintenance studies; deployment-relevant robustness and predeployment safety studies; and governance, implementation, readiness, and human-factors studies. Three themes emerged: trustworthiness after development was conditional and context-dependent; algorithmovigilance extended beyond aggregate performance tracking to include operational, workflow, fairness, contextual, and user-feedback signals; monitoring was more actionable when linked to corrective pathways, governance structures, and institutional readiness. Sociotechnical failures included automation-bias signals, workflow burden, reasoning–conclusion misalignment, and workflow-fit problems. Conclusions: Post-development clinical AI evaluation remains a layered and emerging field rather than a mature monitoring literature. Direct live evidence is limited, concentrated in high-income settings, and weighted towards radiology. The findings should be interpreted as synthesis-informed rather than as empirically validated standards for lifecycle oversight.

## 1. Introduction

Clinical artificial intelligence (AI) has moved rapidly from proof-of-concept development into clinical evaluation, regulatory review, and, increasingly, routine care [1,2]. AI systems are now being introduced across diagnosis, prognosis, triage, workflow prioritisation, and clinical decision support, while trials of AI-assisted interventions are beginning to appear more frequently in the clinical literature [3,4,5]. However, the decisive question for clinical medicine is no longer simply whether an algorithm performs well in retrospective development datasets or internal validation exercises. Rather, it is whether that performance remains reliable, clinically useful, safe, and equitable when the system leaves the development environment and enters intended settings of care [6,7,8].

That distinction is fundamental because clinical AI does not enter a neutral environment. After model development, performance may be reshaped by differences in patient case-mix, data capture, local information systems, workflow design, clinician behaviour, and evolving standards of care [2,9,10]. An apparently high-performing model may therefore deteriorate, miscalibrate, transfer poorly across sites, or generate unintended effects once exposed to real-world clinical conditions. In this sense, the central challenge of clinical AI is not only whether a model can achieve technical accuracy, but whether it can sustain trustworthy performance after translation into practice [11,12,13]. Deployment-related robustness is therefore broader than discrimination alone; it also encompasses calibration, subgroup stability, operational reliability, safety in use, and resilience to changing clinical and organisational contexts [14,15]. For the purposes of this review, deployment-related robustness was operationalised as the capacity of a clinical AI system to maintain safe, reliable, clinically meaningful, and equitable behaviour when evaluated beyond the original development environment and exposed to real or deployment-relevant variation in patients, data, workflows, users, technology, and organisational context.

Many of the most consequential failures emerge only after development. Once implemented, clinical AI systems may encounter data drift, concept drift, calibration decay, hidden failure modes, workflow mismatch, automation bias, alert burden, and feedback loops that alter the conditions under which the model operates [13,16,17,18].

The bridge between model development and full clinical use is particularly important yet remains insufficiently synthesised. Silent or shadow-mode evaluations have increasingly been proposed as an intermediate phase in which AI systems are tested prospectively in the intended clinical environment without influencing patient care [19,20,21]. Bridge-phase evidence is therefore increasingly invoked as part of responsible translation but remains conceptually unsettled and insufficiently integrated with adjacent evidence from prospective and real-world studies.

A similar fragmentation characterises the literature on monitoring and lifecycle oversight after development. Monitoring of clinical AI in routine care is widely advocated, but the empirical evidence on how it is actually operationalised remains limited and methodologically diverse [22,23,24,25]. Review of procurement, integration, monitoring, and evaluation frameworks likewise suggests that downstream action, accountability, and long-term oversight remain less developed than planning and study design [26]. At the same time, algorithmovigilance has been proposed as a useful framework for detecting, understanding, and responding to adverse effects and other risks that emerge during real-world AI use [27]. Read separately, the literature clarifies important parts of the problem. Taken together, however, it does not yet provide an integrated empirical account of how clinical AI is evaluated after model development.

A scoping review is therefore warranted. The relevant literature is methodologically heterogeneous, conceptually diffuse, and dispersed across external validation studies, prospective clinical evaluations, bridge-phase or silent studies, implementation research, monitoring studies, and governance-oriented empirical analyses. Under these conditions, the immediate priority is not a narrowly pooled estimate of effectiveness, but a structured mapping of what has been studied, how evaluation after model development has been operationalised, which dimensions of deployment-related robustness and oversight have been examined, and where the most important evidence gaps remain. Scoping review methodology is particularly appropriate for areas in which concepts are still evolving, terminology is inconsistent, and evidence spans multiple study designs and disciplinary traditions [28,29].

Accordingly, this scoping review aims to map and synthesise the empirical evidence on clinical AI evaluation after model development, focusing on deployment-related robustness, post-development monitoring, and lifecycle oversight in practice. Specifically, the review has two objectives: first, to identify reported risks, unintended consequences, and robustness challenges of clinical AI systems across external, prospective, bridge-phase, and real-world evaluations; and second, to examine how post-development monitoring, updating, auditing, and governance of clinical AI systems have been studied and operationalised in clinical practice. By bringing these currently fragmented strands of evidence into a single analytic frame, this review seeks to clarify how clinical AI behaves after development, how failure and performance change are detected and managed, and where the empirical foundations for safe, effective, and equitable implementation remain insufficient.

## 2. Methods

### 2.1. Study Design

We conducted a scoping review to map and synthesise the empirical literature on clinical artificial intelligence (AI) evaluation after model development, with a particular focus on deployment-related robustness, post-development monitoring, and lifecycle oversight in clinical practice. A scoping review design was selected because the relevant literature is methodologically heterogeneous, conceptually diffuse, and dispersed across multiple forms of evidence, including external validation studies, bridge-phase or silent evaluations, direct post-deployment monitoring studies, methodological studies of drift and updating, and governance- or implementation-oriented empirical analyses [28,30,31]. Under these conditions, the immediate priority is not a narrowly pooled estimate of effect, but a structured mapping of what has been studied, how evaluation after model development has been operationalised, which dimensions of deployment-related robustness and oversight have been examined, and where the most important evidence gaps remain [28,30,31]. Scoping reviews are recommended when the purpose is to map the extent, range, and nature of evidence; clarify concepts and definitions; and identify knowledge gaps in fields where terminology is inconsistent and study designs are diverse [28,30,31].

The review was conducted using Joanna Briggs Institute methodological guidance for scoping reviews and reported in accordance with the Preferred Reporting Items for Systematic Reviews and Meta-Analyses extension for Scoping Reviews (PRISMA-ScR) [28]. PRISMA-ScR is the dedicated reporting framework for scoping reviews and was developed specifically to improve transparency in rationale, eligibility criteria, information sources, search strategy, selection of sources of evidence, data charting, and synthesis reporting [28,32]. Because the review sought not only to map the range of evidence but also to identify recurring patterns across heterogeneous empirical studies, the analytic component of the review used inductive thematic synthesis to develop higher-order themes spanning different study designs and stages of evaluation [28,33].

**Protocol registration and methodological transparency**

A formal review protocol was not registered in PROSPERO, OSF, or another public registry, and no protocol was made publicly available before the conduct of the review. However, the review question, eligibility criteria, information sources, screening process, and data-charting domains were developed a priori before full-text screening and synthesis. This absence of a publicly registered protocol is acknowledged as a methodological transparency limitation.

### 2.2. Eligibility Criteria

Eligibility criteria were developed a priori and were structured around the review concept rather than around a conventional intervention–comparator–outcome framework. Eligibility criteria were developed a priori and were structured around the review concept rather than around a conventional intervention–comparator–outcome framework. The inclusion and exclusion criteria applied during screening and full-text eligibility assessment are summarised in Table 1.

These criteria were intentionally written to match the review question and the actual structure of the field described in the manuscript. In particular, governance, implementation, and usability studies were included only when they contributed empirical evidence relevant to monitoring capacity, organisational oversight, deployment conditions, post-launch feedback, or lifecycle governance. Likewise, predeployment robustness studies were included only when they provided deployment-relevant evidence on transportability, hidden failure modes, reasoning or interpretation failures, or clinician–AI interaction risks directly relevant to the review objectives.

### 2.3. Information Sources

Four bibliographic databases were searched: MEDLINE via PubMed, Embase, Scopus, and Web of Science Core Collection. These databases were selected to capture biomedical, clinical informatics, imaging, implementation, and interdisciplinary literature relevant to clinical AI evaluation after model development. Searches were conducted with no lower date restriction because the purpose of the review was evidence mapping rather than bibliometric trend analysis. The phrase “from inception” refers to the available indexed coverage of each database at the time of searching, rather than to a single shared calendar start date across all databases. Database coverage differed by platform; therefore, the searches should be interpreted as having no lower date limit within each database’s available coverage, with a common upper search date of 28 February 2026.

Only bibliographic metadata required for screening and eligibility assessment were exported from the databases. PubMed/MEDLINE records were accessed through publicly available PubMed search and export functions. Embase, Scopus, and Web of Science Core Collection were accessed through institutional subscriptions and platform-authorised export functions. No full-text mining, redistribution of database content, or commercial reuse of database records was undertaken. No additional ethics approval was required because the review used published bibliographic metadata and published articles; however, access to subscription databases depended on institutional authentication and the relevant database licence conditions.

### 2.4. Search Strategy

A comprehensive search strategy was developed to identify empirical evidence on the post-development evaluation, implementation, monitoring, updating, and governance of artificial intelligence (AI) in healthcare. The strategy combined controlled vocabulary and free-text terms across five conceptual domains: AI and machine learning; healthcare and clinical settings; transition from model development to deployment; post-deployment monitoring, drift, updating, surveillance, and feedback; and governance, implementation, workflow integration, usability, and organisational readiness.

The search strategy was initially developed for MEDLINE via PubMed and subsequently adapted for Embase, Scopus, and Web of Science Core Collection using database-specific subject headings, field tags, truncation, and syntax. Searches were conducted with no lower date restriction within each database’s available indexed coverage and with a common upper search date of 28 February 2026, with the language limited to English. The final database-specific strategies are presented in Table 2. In addition to database searching, backward reference-list screening of included studies and key related reviews was undertaken. Forward citation tracking was conducted using Scopus and Web of Science Core Collection on 28 February 2026. Records identified through citation tracking were screened against the same eligibility criteria used for database-search records, and the results were documented in the PRISMA-ScR flow diagram and Supplementary Table S2.

Grey literature, preprint servers, regulatory databases, vendor documentation, and institutional post-market reports were not systematically searched. This decision was made to preserve a reproducible, peer-reviewed empirical evidence base and to ensure that included sources provided sufficient methodological and results detail for evidence classification and thematic synthesis. However, because governance, implementation, and post-market monitoring evidence may appear outside peer-reviewed journals, this exclusion is acknowledged as a limitation.

### 2.5. Selection of Sources of Evidence

All records retrieved from database searches were imported into Rayyan [34]. Rayyan was used to support screening management, conflict identification, duplicate detection, and reviewer decision tracking. Because automated duplicate detection may miss records with incomplete or inconsistent metadata, Rayyan-assisted duplicate detection was followed by targeted manual verification by the review team. Duplicate and near-duplicate records were checked using combinations of title, author names, publication year, DOI, PMID, journal, and publication status. Manual verification was used to confirm records flagged as potential duplicates and to resolve uncertain cases with missing or inconsistent bibliographic information. Given the moderate number of records after deduplication, this approach was feasible. In substantially larger reviews, duplicate management may require more scalable procedures, such as exact DOI or PMID matching, fuzzy matching of title–author–year combinations, and deduplication functions in review-management platforms such as Covidence, EndNote, Zotero, DistillerSR, or EPPI-Reviewer. In such cases, manual review is usually most practical when limited to ambiguous records.

Title and abstract screening and full-text eligibility assessment were conducted by two reviewers using the prespecified eligibility criteria. Screening was undertaken independently by two reviewers at each stage, with disagreements resolved through discussion and, where necessary, adjudication by a third reviewer. Selection decisions were made against the conceptual scope of the review rather than against an artificially narrow post-deployment-only definition, to ensure consistent handling of bridge studies, methodological monitoring papers, deployment-relevant robustness studies, and governance or implementation studies that were clearly relevant to the review objectives but were not direct live comparative evaluations.

**Figure 1:**
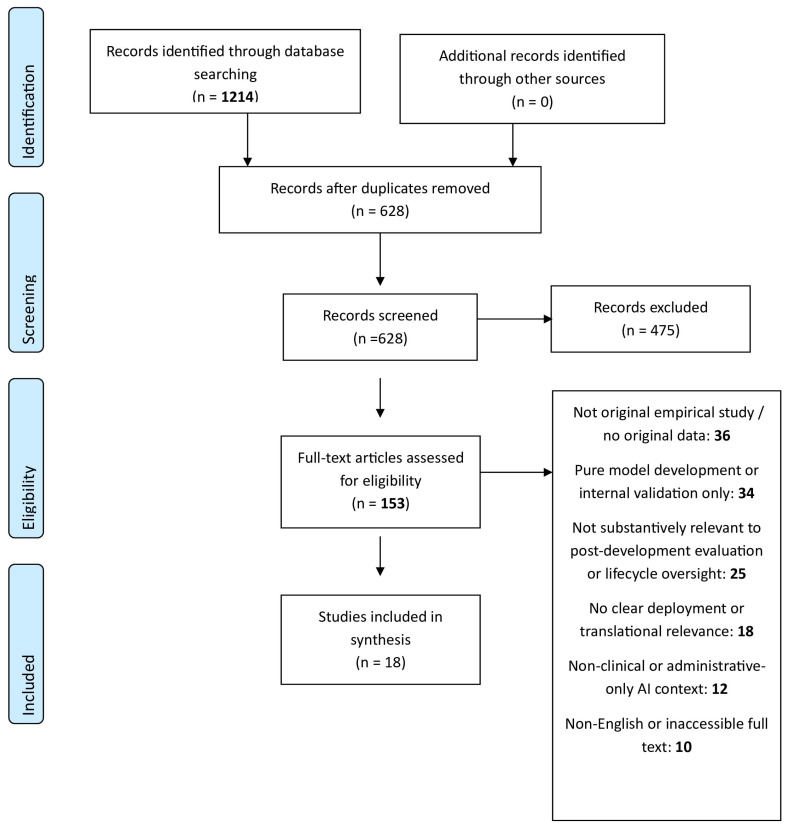
PRISMA flow diagram.

Reasons for exclusion at the full-text stage were recorded. The study-selection process, including title and abstract exclusion categories and full-text exclusion reasons, is reported in the Results section, Figure 1, and Supplementary Table S2. Database-specific record counts, supplementary citation-searching results, deduplication numbers, screened records, full-text assessments, and final inclusion counts are provided in Supplementary Table S2. Although screening was conducted independently by two reviewers, formal inter-rater agreement statistics were not calculated. This was because the review used an iterative scoping approach in a conceptually heterogeneous field, and disagreements were resolved through discussion and consensus rather than quantified as a separate reliability outcome. The absence of formal agreement statistics is acknowledged as a reproducibility limitation.

### 2.6. Data Charting Process

A standardised data-charting form was developed and iteratively refined to ensure consistent extraction across the heterogeneous study designs represented in the review. The final data-charting form is provided as Supplementary Table S1.

The form captured author and year; country and setting; clinical domain; AI modality; study design; population or data source; deployment phase; evidence stratum; whether outputs were used to guide care; deployment or workflow context; monitoring or robustness focus; reported performance, safety, operational, fairness, or workflow indicators; type of failure or instability reported; governance or corrective-response mechanisms; key findings; limitations; and relevance to the review objectives.

Data charting was undertaken by two reviewers. Extracted information was compared and reconciled through discussion. Where uncertainty remained about evidence-stratum assignment or interpretation of a study’s deployment relevance, the issue was discussed within the review team until consensus was reached. This approach enabled the review to combine structured evidence mapping with interpretive synthesis of cross-cutting patterns [28,33].

### 2.7. Analytic Framework for Evidence Classification

Included studies were classified into one of five analytically distinct evidence strata according to their dominant empirical contribution. These strata were specified at a conceptual level to organise synthesis and to prevent conflation of direct post-deployment evidence with adjacent deployment-relevant evidence and were operationalised iteratively during charting and synthesis as familiarity with the literature deepened. The framework was therefore used as an analytic scaffold for interpretation rather than as a post hoc substitute for study description. The five evidence strata were used as an analytical tool to organise heterogeneous evidence and calibrate inference. They should not be interpreted as a validated taxonomy of the field, a formal hierarchy of evidence, or a prescriptive implementation model. Their purpose was to prevent evidentiary overcompression by distinguishing direct live monitoring evidence from adjacent forms of deployment-relevant evidence. The operational definitions of the five analytic evidence strata are presented in Table 3.

These strata were used prospectively in the synthesis to prevent evidentiary inflation. Governance, implementation, and usability papers were therefore interpreted primarily as contextual and enabling evidence for Objective 2 rather than as direct proof of model-level monitoring effectiveness. Similarly, deployment-relevant robustness studies were treated as strong evidence for Objective 1 and as indirect or contextual evidence for Objective 2 unless they directly addressed surveillance, maintenance, or updating logic.

### 2.8. Critical Appraisal

No formal risk-of-bias tool was used to exclude studies because the purpose of the review was to map and characterise an emerging and heterogeneous evidence field rather than to estimate pooled effectiveness across comparable studies. Contemporary scoping review guidance treats critical appraisal as contingent on review purpose rather than universally mandatory [28,31,35]. Instead, study design, evidence stratum, and degree of direct post-deployment evidence were explicitly charted and then used to calibrate interpretation.

### 2.9. Synthesis of Results

Given the heterogeneity of the included literature, findings were synthesised in two complementary stages. First, we undertook descriptive evidence mapping of the included studies, summarising their study design, clinical setting, AI application, deployment context, relationship to the review objectives, and position within the analytic evidence-strata framework. This stage was used to clarify the evidentiary proximity of each study to live clinical deployment and to distinguish direct post-deployment monitoring evidence from bridge-phase, methodological, deployment-relevant robustness, and governance or implementation evidence.

Second, we conducted an inductive thematic synthesis of the extracted findings. Findings relevant to deployment-related robustness, monitoring, updating, governance, safety, workflow integration, human–AI interaction, and organisational readiness were extracted from each included study. These extracted findings were then coded line by line using an inductive approach. Related codes were grouped into descriptive themes, and these descriptive themes were iteratively refined into higher-order analytical themes through constant comparison across studies and evidence strata [33]. Coding decisions, evidence-stratum assignments, and the developing theme structure were reviewed iteratively by the review team. Disagreements regarding coding, interpretation, or evidence-stratum assignment were resolved through discussion until consensus was reached.

At all stages, direct post-deployment evidence was distinguished from near-live, methodological, deployment-relevant robustness, and governance or implementation evidence, and the strength of inference was calibrated accordingly. This distinction was essential because direct empirical evidence from live clinical use remains limited, and broader interpretation necessarily depends partly on bridge studies, methodological monitoring work, deployment-relevant robustness studies, and governance or implementation evidence rather than only on mature post-deployment comparative evaluations. No dedicated qualitative data-analysis software, such as NVivo, ATLAS.ti, or MAXQDA, was used. Generative AI tools were not used for study design, study selection, eligibility assessment, data extraction, evidence-stratum assignment, coding, theme generation, analysis, interpretation of findings, citation verification, or the generation of scientific content.

## 3. Results

### 3.1. Study Selection

Database searches identified 1214 records, and supplementary backward and forward citation tracking identified no additional records. After Rayyan-assisted and manual deduplication, 586 duplicate records were removed, leaving 628 records for title and abstract screening. During title and abstract screening, 475 records were excluded because they were not relevant to the review objectives, focused only on model development or internal validation, addressed non-clinical AI, were commentaries, reviews, or protocols, or lacked empirical deployment relevance. Of the 153 full-text articles assessed for eligibility, 135 were excluded: not original empirical studies or no original data (*n* = 36), pure model development or internal validation only (*n* = 34), not substantively relevant to post-development evaluation or lifecycle oversight (*n* = 25), no clear deployment or translational relevance (*n* = 18), non-clinical or administrative-only AI context (*n* = 12), or non-English or inaccessible full text (*n* = 10). Eighteen studies were included in the final synthesis (Figure 1; Supplementary Table S2).

### 3.2. Characteristics of the Included Studies

Eighteen empirical studies and empirically grounded implementation or monitoring reports were included in the final synthesis [11,19,36,37,38,39,40,41,42,43,44,45,46,47,48,49,50,51]. The small final number of included studies reflected the focused eligibility criteria and the emerging nature of the field: many retrieved records addressed model development, internal validation, technical benchmarking, commentaries, protocols, conceptual frameworks, or general AI implementation without empirical post-development evaluation, monitoring, updating, governance, or deployment-relevant robustness evidence. The included literature was heterogeneous in design, deployment context, and evidentiary proximity to routine clinical use. For analytic purposes, studies were organised into five evidence strata: direct live or post-deployment monitoring studies; near-live bridge studies; methodological monitoring and maintenance studies; deployment-relevant robustness and predeployment safety studies; and governance, implementation, readiness, and human-factors studies. Although these strata were analytically distinct, the boundary between bridge evaluation and early operational surveillance was not always sharp in individual studies.

Most studies were conducted in high-income settings. The corpus included studies from the USA, Canada, the UK, the Netherlands, Australia, and China. Clinical applications were broad, but the evidence base was weighted towards radiology and imaging, including intracranial haemorrhage detection, chest radiography, fracture detection, skin lesion assessment, and radiology impression generation. Non-imaging applications included oncology survival prediction, inpatient mortality prediction, blood-culture stewardship, paediatric hydronephrosis prediction, EHR-embedded information retrieval and summarisation, and health-system governance or implementation studies (Appendix A).

### 3.3. Thematic Synthesis

Line-by-line coding of the extracted findings generated three analytical themes, each comprising three descriptive subthemes. These themes cut across the analytic evidence strata, although they were supported by different forms of evidence to different degrees.

Table 4 summarises the three analytical themes, the main evidence signals supporting each theme, and the corresponding interpretation for post-development clinical AI governance.

Theme 1. Conditional trustworthiness after development

This theme captured the recurring pattern that trustworthiness after model development was not fixed, but remained dependent on evaluation context, local conditions, and the form of clinical introduction.

Subtheme 1.1. Performance discontinuity across evaluation contexts

Across the bridge and deployment-relevant robustness studies, strong retrospective or internal performance frequently did not persist under prospective, external, or deployment-relevant evaluation. Kwong et al. (2022) [19] reported marked deterioration at the first prospective silent-trial evaluation. Zech et al. (2018) [44] showed degradation across hospital systems, and Oakden-Rayner et al. (2022) [41] reported threshold instability despite high apparent discrimination. In the LLM studies, strong benchmark or reader-study performance coexisted with safety-relevant residual errors and wrongly altered decisions [37,38]. Across these studies, performance estimates varied materially by evaluation context.

Subtheme 1.2. Heterogeneous sources of instability

Across the included studies, performance change was linked to multiple forms of shift, including event-rate, case-mix, predictor–outcome, hardware, assay, metadata, and workflow changes. Chi et al. (2022) [51] linked deterioration to event-rate, case-mix, and predictor–outcome shifts. Kore et al. (2024) [40] identified clinically meaningful data drift without obvious aggregate deterioration. Subasri et al. (2025) [11] identified harmful shifts related to hospital type, admission source, age distribution, assay changes, and the COVID-19 period. Rohren et al. (2025) [50] linked false-positive variation to scanner manufacturer and artifacts, and Cook et al. (2026) [47] described instability associated with routing changes, image characteristics, and technical architecture. Schinkel et al. (2023) [48], by contrast, reported stable monitored performance despite changing clinical context.

Subtheme 1.3. Sociotechnical manifestations of failure

Failure extended beyond reduced average accuracy. Oakden-Rayner et al. (2022) [41] identified hidden failure modes in abnormal bone and joint cases. Zech et al. (2018) [44] showed that apparent performance could partly reflect institution and prevalence confounding. Rohren et al. (2025) [50] documented a probable automation-bias event. Wang et al. (2025) [38] described reasoning–conclusion misalignment, and Wang et al. (2026) [37] showed that AI assistance could both improve average performance and wrongly alter some decisions. Cook et al. (2026) [47] reported workflow burden associated with technical failure, and Schreier et al. (2025) [45] identified onboarding and workflow-fit issues after deployment.

Theme 2. Algorithmovigilance beyond performance tracking

This theme captured the shift from viewing monitoring as occasional metric review to treating it as broader surveillance of the model, its context, and its downstream operational effects.

Subtheme 2.1. Insufficiency of aggregate metrics

Through several studies, aggregate performance metrics were incomplete monitoring signals. Chi et al. (2022) [51] showed that calibration could deteriorate while discrimination remained relatively stable. Kore et al. (2024) [40] reported that clinically meaningful drift could occur without marked change in aggregate performance. Kim et al. (2025a) [49] showed that feedback loops could make successfully deployed models appear to worsen when observed outcomes had been altered by model-triggered interventions. Across these studies, headline metrics alone did not fully characterise post-development reliability.

Subtheme 2.2. Multimodal surveillance signals

The strongest monitoring-oriented studies combined technical, operational, and contextual signals. Schinkel et al. (2023) [48] combined model metrics with positivity rates, contamination rates, admissions, and case-mix indicators. Rohren et al. (2025) [50] combined report-based surveillance, adjudication, and contextual analysis of scanner- and artifact-related false positives. Thomas et al. (2023) [46] incorporated second-read review, repeat-presentation tracking, root-cause analysis, and software-version comparison. Cook et al. (2026) [47] monitored utilisation, routing failures, algorithmic and technical failures, acquisition characteristics, and user feedback. Salwei et al. (2025) [43] and Kim et al. (2025b) [39] extended this into broader monitoring-system and governance designs spanning performance, process, outcomes, fairness, and user feedback.

Subtheme 2.3. Staged evaluation as controlled observability

Several studies used silent trials, shadow mode, or staged exposure to observe model behaviour before or during constrained clinical introduction. Kwong et al. (2022) [19] evaluated a model prospectively in silent mode before clinician-facing use. Schinkel et al. (2023) [48] monitored a deployed model in real time while withholding outputs from clinicians. Ramsay et al. (2025) [42] reported shadow mode as part of early NHS implementation pathways. Thomas et al. (2023) [46] used a staged post-market model with consultant second-read review and structured false-negative surveillance.

Theme 3. Actionable oversight and institutional readiness

This theme captured the recurring finding that monitoring was more actionable when linked to corrective pathways, governance structures, and local capacity to interpret and act on signals.

Subtheme 3.1. Coupling monitoring to corrective pathways

Several studies described monitoring as linked to maintenance, mitigation, or redesign rather than passive observation alone. Chi et al. (2022) [51] linked surveillance to iterative updating. Kim et al. (2025a) [49] showed that feedback-aware monitoring could avoid harmful retraining decisions. Subasri et al. (2025) [11] linked shift detection to transfer learning and drift-triggered continual learning. Schinkel et al. (2023) [48] used surveillance to judge whether recalibration or correction was warranted. Cook et al. (2026) [47] described routing-rule modification, troubleshooting, and model replacement, and Thomas et al. (2023) [46] described second-read review, root-cause analysis, and post-update surveillance.

Subtheme 3.2. Governance as interpretive infrastructure

Across governance and implementation studies, monitoring required ownership, escalation routes, documentation, committee structures, and lifecycle management. Salwei et al. (2025) [43] designed a monitoring platform around performance, process, outcomes, fairness, and consultation pathways. Kim et al. (2025b) [39] described the creation of governance structures to address fragmented oversight. Ramsay et al. (2025) [42] showed that local governance variation shaped implementation and evaluation. Hwang et al. (2026) [36] showed heterogeneity in institutional monitoring and evaluation practices. Direct operational studies also embedded monitoring within structured review and interdisciplinary oversight [46,47].

Subtheme 3.3. Readiness inequities in lifecycle oversight

The ability to sustain monitoring was uneven across organisations. Hwang et al. (2026) [36] showed that hospitals differed substantially in reported evaluation and post-implementation monitoring practices. Ramsay et al. (2025) [42] identified staffing, contracting, interoperability, project-management capacity, and data linkage as implementation constraints. Schreier et al. (2025) [45] showed that successful post-launch use depended on training, workflow integration, and user-centred evaluation. Thomas et al. (2023) [46] also indicated that subgroup-sensitive post-market surveillance required continuing pathway-level infrastructure.

Taken together, the included studies did not yield a single mature empirical evidence base of post-development clinical AI monitoring in routine care. Rather, they described a limited direct core of live or near-live monitoring evidence, surrounded by methodological studies explaining how monitoring might need to work, deployment-relevant robustness studies showing why monitoring is needed, and governance and implementation studies indicating what conditions may be required to sustain it. Across these strands of evidence, three analytical patterns recurred: trustworthiness after development varied by context, surveillance extended beyond performance tracking, and monitoring was most often linked to corrective pathways and institutional capacity.

## 4. Discussion

This review suggests that the central challenge of clinical AI after model development is not singular. Rather, trustworthy translation appears to depend on the interaction between two linked problems: residual technical fragility before or during early clinical introduction, and uneven institutional capacity to monitor, interpret, and govern systems after introduction. The review therefore does not support a simple transition from a model-quality problem to a governance-after-deployment problem. Instead, it indicates that robustness, surveillance, and oversight remain interdependent components of post-development evaluation in practice.

A second overarching interpretation is that the direct empirical basis for live post-deployment monitoring remains limited. The strongest operational evidence came from a small number of studies, was concentrated in high-income settings, and was weighted towards radiology and other technically mature services. Much of the broader synthesis therefore depends on bridge studies, methodological work, deployment-relevant robustness studies, and governance or implementation evidence rather than on direct post-deployment comparative outcome evaluations. That limitation should shape the strength of inference drawn from this review. The most defensible interpretation is not that a mature post-deployment monitoring paradigm has already been established, but that the empirical foundations of trustworthy clinical AI after development remain incomplete and unevenly distributed.

The included LLM and large-reasoning-model studies were classified as deployment-relevant robustness and predeployment safety studies rather than as direct monitoring evidence [37,38]. These studies differ from traditional predictive ML studies because their outputs are generative, linguistic, and reasoning-mediated rather than limited to fixed risk scores or classifications. The large-reasoning-model radiology study reported reasoning–conclusion misalignment, including cases where correct reasoning led to an incorrect final conclusion [38]. The brain MRI LLM study reported improved diagnostic performance and reading efficiency, but also non-trivial incorrect alterations and risk of propagation of LLM-generated errors [37]. These risks map onto the broader robustness framework through the dimensions of human–AI interaction, sociotechnical safety, operational reliability, and correctability. Their inclusion therefore broadens the review’s account of post-development fragility, but does not provide evidence of live post-deployment monitoring effectiveness. For this reason, LLM studies were interpreted as predeployment safety and robustness evidence with implications for lifecycle oversight, rather than as proof of mature algorithmovigilance in routine care [37,38].

More broadly, the review suggests that trustworthy clinical AI after development depends on five closely linked conditions: context-sensitive robustness assessment before and during introduction into care; cautious or staged entry into clinical environments when uncertainty remains high; surveillance that extends beyond headline performance metrics to include operational, workflow, and contextual signals; defined response pathways through which concerning signals can trigger review, recalibration, retraining, route modification, or additional human oversight; and governance arrangements capable of sustaining accountability, escalation, lifecycle management, and equity-sensitive evaluation. These domains should therefore be understood as recurring interpretive patterns across heterogeneous evidence, rather than as a prescriptive implementation sequence or a validated oversight model.

The present synthesis is broadly consistent with the wider literature. In their scoping review, Andersen et al. (2024) [24] concluded that research on monitoring clinical AI is sparse and heterogeneous. That aligns closely with the present review, particularly the finding that only a small subset of included studies provided direct live or near-live monitoring evidence, whereas much of the surrounding literature consisted of methodological, governance, implementation, or deployment-relevant robustness studies rather than mature post-deployment evaluations in practice. Our findings are therefore consistent with Andersen et al. (2024) [24], but expand upon this work by showing more explicitly that the apparent monitoring literature is a layered field with different evidentiary strata rather than a single empirical body of work.

Our interpretation is also consistent with the emerging algorithmovigilance literature. Balendran et al. (2024) [27] argued that post-deployment AI oversight should learn from pharmacovigilance because adverse effects and operational risks can emerge after real-world introduction. The direct monitoring studies in our review support that orientation, particularly where operational failures, false positives, routing breakdowns, workflow harms, and possible automation bias appeared after clinical introduction. At the same time, the present synthesis suggests a necessary caution: the empirical base underpinning algorithmovigilance in healthcare remains thin and is still heavily supplemented by conceptual or framework-oriented work.

The findings also align with the external literature on silent trials. In their scoping review, Tikhomirov et al. (2026) [21] identified substantial heterogeneity in silent-trial terminology and reporting and noted the absence of formal guidance for silent AI evaluation in healthcare. That is highly consistent with the present review. Kwong et al. (2022) [19] and, in a different form, Schinkel et al. (2023) [48] support the view that silent or shadow-mode evaluation can play an important transitional role between retrospective validation and unrestricted live use but also show that this phase remains methodologically variable and not yet standardised.

Our governance and implementation findings also converge with the broader framework literature. In their systematic review of frameworks, Khan et al. (2024) [26] concluded that implementation science for clinical AI remains operationally immature and that consensus on how AI tools should be introduced, maintained, and evaluated in practice is still limited. That pattern closely mirrors the present synthesis. Our included studies, governance, readiness, and implementation work repeatedly described the need for ownership, reporting structures, feedback channels, and decommissioning logic, yet direct empirical evidence showing how organisations actually monitor, adapt, and retire models in routine practice remained limited.

The present review is also consistent with the broader RCT literature, although that literature addresses a somewhat different question. Han et al. (2024) [52] documented in their scoping review a rapidly growing trial literature on AI in clinical practice, but it did not establish a comparable evidence base for post-deployment monitoring architectures or adaptive governance models. Our review therefore complements rather than duplicates the RCT literature: where Han et al. (2024) [52] map interventional evidence on AI tools in practice, the present synthesis highlights that comparable interventional evidence for monitoring systems, response pathways, and post-deployment governance arrangements remains largely absent.

Finally, our cautious interpretation is reinforced by the National Institute of Standards and Technology (NIST) Artificial Intelligence Risk Management Framework, which emphasises lifecycle-oriented risk management across the design, development, use, and evaluation of AI systems [53]. Applied to post-development clinical AI, this perspective supports the need for continuing governance, measurement, monitoring, and management processes after deployment. One of the principal contributions of our synthesis is to show why fragmentation persists in this field: direct post-deployment studies, bridge evaluations, methodological drift and updating studies, robustness studies, and governance or implementation papers are often discussed together, even though they differ substantially in evidentiary reach.

### Interpretive Framework for Inference in Clinical AI Deployment Studies

A central problem in this literature is that studies with very different evidentiary reach are often discussed together under a common language of clinical AI evaluation. To improve interpretive precision, we propose an interpretive framework for inference in clinical AI deployment studies, clarifying what different study designs can reasonably support regarding claims about deployment, monitoring, and governance. The framework is not a formal hierarchy of evidence and is not intended to rank study quality, and should not be interpreted as a validated model of implementation. Rather, it is a heuristic designed to distinguish the inferential reach of development-stage, transportability, bridge-phase, direct operational, methodological monitoring, and contextual implementation evidence. The proposed interpretive framework for inference in clinical AI deployment studies is summarised in Table 5.

This framework matters because one of the major risks in this field is rhetorical overcompression: studies of very different design, purpose, and evidentiary reach are often invoked together as though they support identical claims about clinical deployment. The present review suggests that such flattening obscures both what is known and what remains uncertain. A more careful distinction between transportability, bridge-phase, direct operational, methodological, and contextual implementation evidence is therefore necessary if post-development evaluation is to mature as a field rather than remain a loose assemblage of adjacent literature.

Implications for practice, health systems, and future research

The implications of this review are best understood as synthesis-informed rather than as empirically validated standards. The available evidence suggests that trustworthy clinical translation is unlikely to be achieved through retrospective performance alone and that post-development surveillance cannot be reduced to periodic discrimination metrics. Instead, the literature supports a more cautious model in which context-sensitive robustness assessment, staged introduction when uncertainty remains high, and broader monitoring of operational, workflow, technical, and subgroup-sensitive signals are treated as prudent components of responsible deployment, even though comparative evidence for any one surveillance architecture remains limited.

For health systems, a cautious implication is that monitoring is unlikely to be sustainable without ownership, data infrastructure, reporting channels, escalation routes, and some capacity for action when concerning signals arise. For research, the clearest priority is direct comparative evidence: studies that test whether particular monitoring architectures, staged deployment strategies, or governance-linked response systems improve patient, clinician, or health-system outcomes. Broader evidence is also needed outside radiology, outside high-income settings, and outside technically mature organisations.

Strengths and limitations

A strength of this review is that it synthesised direct monitoring, bridge-phase, methodological, robustness, and governance evidence while preserving distinctions in evidentiary reach. By doing so, it offers a more realistic account of what is and is not currently known than would be possible through a narrower account of post-deployment monitoring alone. A further strength is that the review explicitly sought to avoid evidentiary inflation by separating model-level operational evidence from contextual governance or implementation evidence.

The review also has important limitations. Direct empirical evidence from live clinical use remains sparse, high-income, radiology-heavy, observational, and often institution-specific. Many of the broader conclusions, therefore, depend on supplementary evidence strata rather than on direct post-deployment comparative studies. Prospective interventional trials were absent. Governance and implementation studies were informative about conditions and structures, but did not provide direct proof of model-level monitoring effectiveness. Methodological studies were crucial for explaining surveillance logic, but were not substitutes for live clinical outcome evaluations. Because grey literature, preprint servers, regulatory databases, vendor documentation, and institutional post-market reports were not systematically searched, this review may have missed deployment and monitoring evidence that remains outside the peer-reviewed journal literature. Because bibliographic databases are continuously updated and differ in indexing coverage, document-type classification, early-access handling, and export functions, rerunning the same search strategy at a later date may yield record counts that differ from those reported in the PRISMA-ScR flow diagram and Supplementary Table S2. These limitations mean that the review should be interpreted as a structured synthesis of an emerging field rather than as a definitive empirical statement that one post-deployment monitoring model has already been validated.

## 5. Conclusions

This review suggests that trustworthy clinical AI, after development, remains constrained by a dual problem: technical fragility may persist before and during early clinical introduction, and institutional capacity to monitor and govern systems after introduction remains uneven and underdeveloped. The direct empirical evidence for live post-deployment monitoring is still limited. Therefore, the implications of this review should be understood as synthesis-informed rather than as empirically validated standards. The wider surrounding literature nevertheless indicates why monitoring is needed, what it may need to account for, and what organisational conditions may be required to sustain it. The most defensible conclusion is therefore not that the field has already established a mature post-deployment monitoring paradigm, but that robust clinical translation may require closer integration of context-sensitive robustness assessment, cautious introduction into care, broader surveillance architectures, defined response pathways, and accountable governance.

## Figures and Tables

**Table 1:** Eligibility criteria used in the review.

Domain	Inclusion Criteria	Exclusion Criteria
**Population and setting**	Clinical AI systems, machine-learning models, deep-learning systems, large language models, AI-enabled clinical decision support, or organisational AI oversight in human healthcare settings	Non-clinical AI applications; administrative, financial, or operational algorithms without direct clinical relevance
**Core review concept**	Empirical evaluation after model development, including direct post-deployment evaluation, bridge-phase or silent/shadow-mode evaluation, methodological monitoring or updating studies, deployment-relevant robustness studies, and empirical governance, implementation, readiness, or usability studies directly relevant to lifecycle oversight	Pure model-development studies; internal-validation-only studies; technical benchmark papers without deployment or translational relevance; studies focused only on model construction
**Study type**	Empirical studies and empirically grounded implementation or monitoring reports reporting original quantitative, qualitative, mixed-methods, operational, design, or evaluative data	Editorials, commentaries, letters, narrative reviews, protocols, consensus statements, conference abstracts without sufficient data, and papers without original empirical content
**Relevance to review objectives**	Studies addressing at least one of the two review objectives: (1) risks, unintended consequences, or robustness challenges after development; and/or (2) post-development monitoring, updating, auditing, governance, or lifecycle oversight	Studies not substantively relevant to either objective
**Deployment relevance**	Studies providing direct or adjacent deployment-relevant evidence, including external validation with translational relevance, post-deployment monitoring, bridge evaluation, methodological maintenance, or organisational oversight of clinical AI	Studies with no clear relevance to translation into practice or lifecycle evaluation
**Publication type**	Full-text, peer-reviewed journal articles	Abstract-only reports, duplicate publications, inaccessible full texts
**Language**	English	Non-English publications
**Time frame**	No lower date restriction within each database’s available indexed coverage; common upper search date of 28 February 2026	Publications after 28 February 2026

**Table 2:** Full database search strategies.

Database	Search String
**MEDLINE (PubMed)**	((“Artificial Intelligence”[Mesh] OR “Machine Learning”[Mesh] OR artificial intelligence[tiab] OR machine learning[tiab] OR deep learning[tiab] OR neural network*[tiab] OR “large language model*”[tiab] OR LLM*[tiab] OR “predictive model*”[tiab]) AND (“Delivery of Health Care”[Mesh] OR “Clinical Decision Support Systems”[Mesh] OR clinical[tiab] OR healthcare[tiab] OR “health care”[tiab] OR hospital*[tiab] OR clinic*[tiab] OR radiolog*[tiab] OR dermatolog*[tiab] OR oncology[tiab] OR emergency[tiab] OR “electronic health record*”[tiab] OR EHR[tiab]) AND (“external validation”[tiab] OR “temporal validation”[tiab] OR “prospective evaluat*”[tiab] OR “silent trial*”[tiab] OR “shadow mode”[tiab] OR “bridge phase”[tiab] OR “bridge-phase”[tiab] OR implementation[tiab] OR deployment[tiab] OR “post-deployment”[tiab] OR postdeployment[tiab] OR “post-market”[tiab] OR postmarket[tiab] OR monitoring[tiab] OR surveillance[tiab] OR audit*[tiab] OR algorithmovigilance[tiab] OR drift[tiab] OR “data shift”[tiab] OR “dataset shift”[tiab] OR “concept drift”[tiab] OR “calibration drift”[tiab] OR recalibrat*[tiab] OR retrain*[tiab] OR “model updat*”[tiab] OR “continual learning”[tiab] OR “feedback loop*”[tiab] OR governance[tiab] OR oversight[tiab] OR workflow[tiab] OR usability[tiab] OR adoption[tiab] OR readiness[tiab])) AND (“1 January 1900” [Date—Publication]: “28 February 2026” [Date—Publication]) AND English[lang]
**Embase**	(‘artificial intelligence’/exp OR ‘machine learning’/exp OR ‘deep learning’/exp OR ‘artificial intelligence’:ti,ab,kw OR ‘machine learning’:ti,ab,kw OR ‘deep learning’:ti,ab,kw OR ‘large language model*’:ti,ab,kw OR ‘predictive model*’:ti,ab,kw) AND (‘health care’/exp OR ‘clinical decision support system’/exp OR clinical:ti,ab,kw OR hospital*:ti,ab,kw OR clinic*:ti,ab,kw OR radiolog*:ti,ab,kw OR dermatolog*:ti,ab,kw OR oncology:ti,ab,kw OR emergency:ti,ab,kw OR ‘electronic health record*’:ti,ab,kw OR EHR:ti,ab,kw) AND (‘external validation’:ti,ab,kw OR ‘temporal validation’:ti,ab,kw OR ‘prospective evaluat*’:ti,ab,kw OR ‘silent trial*’:ti,ab,kw OR ‘shadow mode’:ti,ab,kw OR ‘bridge phase’:ti,ab,kw OR implementation:ti,ab,kw OR deployment:ti,ab,kw OR ‘post deployment’:ti,ab,kw OR ‘post market’:ti,ab,kw OR monitoring:ti,ab,kw OR surveillance:ti,ab,kw OR audit*:ti,ab,kw OR algorithmovigilance:ti,ab,kw OR drift:ti,ab,kw OR ‘data shift’:ti,ab,kw OR ‘dataset shift’:ti,ab,kw OR ‘concept drift’:ti,ab,kw OR ‘calibration drift’:ti,ab,kw OR recalibrat*:ti,ab,kw OR retrain*:ti,ab,kw OR ‘model updating’:ti,ab,kw OR ‘continual learning’:ti,ab,kw OR ‘feedback loop*’:ti,ab,kw OR governance:ti,ab,kw OR oversight:ti,ab,kw OR workflow:ti,ab,kw OR usability:ti,ab,kw OR adoption:ti,ab,kw OR readiness:ti,ab,kw) AND [humans]/lim AND [english]/lim AND [1900–2026]/py
**Scopus**	TITLE-ABS-KEY((“artificial intelligence” OR “machine learning” OR “deep learning” OR “large language model*” OR LLM* OR “predictive model*”) AND (clinical OR healthcare OR “health care” OR hospital* OR clinic* OR radiolog* OR dermatolog* OR oncology OR emergency OR “electronic health record*” OR EHR) AND (“external validation” OR “temporal validation” OR “prospective evaluation” OR “silent trial*” OR “shadow mode” OR “bridge phase” OR implementation OR deployment OR “post-deployment” OR postdeployment OR “post-market” OR postmarket OR monitoring OR surveillance OR audit* OR algorithmovigilance OR drift OR “data shift” OR “dataset shift” OR “concept drift” OR “calibration drift” OR recalibrat* OR retrain* OR “model updating” OR “continual learning” OR “feedback loop*” OR governance OR oversight OR workflow OR usability OR adoption OR readiness)) AND PUBYEAR < 2027 AND (LIMIT-TO(LANGUAGE, “English”))
**Web of Science Core Collection**	TS = ((“artificial intelligence” OR “machine learning” OR “deep learning” OR “large language model*” OR LLM* OR “predictive model*”) AND (clinical OR healthcare OR “health care” OR hospital* OR clinic* OR radiolog* OR dermatolog* OR oncology OR emergency OR “electronic health record*” OR EHR) AND (“external validation” OR “temporal validation” OR “prospective evaluation” OR “silent trial*” OR “shadow mode” OR “bridge phase” OR implementation OR deployment OR “post-deployment” OR postdeployment OR “post-market” OR postmarket OR monitoring OR surveillance OR audit* OR algorithmovigilance OR drift OR “data shift” OR “dataset shift” OR “concept drift” OR “calibration drift” OR recalibrat* OR retrain* OR “model updating” OR “continual learning” OR “feedback loop*” OR governance OR oversight OR workflow OR usability OR adoption OR readiness)) Refined by: DOCUMENT TYPES = (ARTICLE OR EARLY ACCESS); Timespan: no lower date restriction to 28 February 2026; Languages: English

**Table 3:** Operational definitions of the analytic evidence strata.

Evidence Stratum	Operational Definition
**Direct live or post-deployment monitoring studies**	Studies evaluating AI systems after introduction into clinical workflows or routine service use
**Near-live bridge studies**	Prospective evaluations in intended clinical environments in which outputs were generated in real time but did not guide patient care, including silent-trial and shadow-mode studies
**Methodological monitoring and maintenance studies**	Empirical studies of drift detection, recalibration, retraining, continual learning, or feedback-aware surveillance intended to support post-development monitoring
**Deployment-relevant robustness and predeployment safety studies**	External validation, audit, or robustness studies with clear implications for deployment-related fragility, hidden failure modes, transportability, or clinician–AI interaction risk
**Governance, implementation, readiness, and human-factors studies**	Empirical studies of organisational oversight, monitoring infrastructure, implementation conditions, usability, adoption, training needs, and post-launch feedback relevant to lifecycle oversight

**Table 4:** Summary of key findings from the thematic synthesis.

Analytical Theme	Main Finding	Key Evidence Signals	Interpretation
Conditional trustworthiness after development	Clinical AI trustworthiness was not fixed after model development and varied across evaluation context, patient mix, local data, workflows, and deployment conditions.	Prospective silent-trial deterioration, external performance degradation, threshold instability, hidden failure modes, automation-bias signals, workflow burden, and LLM reasoning–conclusion misalignment.	Robustness should be assessed before and during introduction into care, not inferred from internal validation alone.
Algorithmovigilance beyond performance tracking	Monitoring required more than periodic review of aggregate discrimination or accuracy metrics.	Calibration drift, clinically meaningful data shift without obvious aggregate deterioration, scanner/protocol effects, routing failures, user feedback, false-positive surveillance, and feedback-loop bias.	Effective surveillance should combine technical, operational, workflow, contextual, fairness, and user-feedback signals.
Actionable oversight and institutional readiness	Monitoring was more meaningful when linked to corrective pathways, governance structures, and institutional capacity.	Recalibration, retraining, second-read review, root-cause analysis, routing-rule modification, governance committees, escalation routes, and readiness gaps across organisations.	Post-development evaluation requires institutional ownership, response pathways, documentation, and capacity to act on monitoring signals

**Table 5:** Interpretive framework for inference in clinical AI deployment studies.

Inference Category	Typical Study Type	What It Can Reasonably Support	What It Cannot by Itself Establish
**Development-stage inference**	Internal retrospective validation	Initial model performance in development data	Real-world transportability, monitored performance, or safe routine use
**Transportability inference**	External or temporal validation; algorithmic audit	Whether performance, thresholds, or behaviour vary across settings or time	Safe routine clinical use after deployment
**Bridge-phase inference**	Silent trial; shadow mode	Real-time local behaviour before unrestricted clinician-facing use	Full clinician–AI interaction effects in routine care
**Direct operational monitoring inference**	Live post-deployment or post-market surveillance	That monitoring can detect operational or clinical signals in practice	Comparative proof that one monitoring architecture improves outcomes
**Methodological monitoring inference**	Simulation; drift detection; continual learning	Why monitoring may fail, what signals may matter, or how surveillance might be designed	Actual effectiveness of those strategies in live care
**Contextual implementation and governance inference**	Governance, readiness, design, or implementation studies	What organisational conditions may enable or constrain monitoring and oversight	Direct model-level post-deployment safety outcomes

## Data Availability

No new data were created or analysed in this study.

## Supplementary Materials

The following supporting information can be downloaded at https://www.mdpi.com/article/10.3390/healthcare14142052/s1, Table S1: Standardised data-charting form used for extraction across included studies; Table S2: Database-specific search results, citation-tracking results, deduplication records, screening outcomes, full-text exclusion reasons, and final inclusion counts. Table S3: Preferred Reporting Items for Systematic Reviews and Meta-Analyses extension for Scoping Reviews (PRISMA-ScR) Checklist.

## Appendix A. Characteristics of Included Studies

**Study**	**Setting and AI Use**	**Evidence Phase**	**Study Design and Scale**	**Deployment/Workflow Context**	**Monitoring Focus**	**Principal Findings**	**Key Limitations**
Rohren et al., 2025 [50]	USA; multicentre radiology network; AI-assisted intracranial haemorrhage detection on head CT with LLM-supported auditing	Live post-deployment evaluation	Retrospective multicentre monitoring study; 332,809 examinations; adjudicated sample of 200 AI-positive cases	Aidoc operating in routine workflow; ChatGPT-4 Turbo used as a monitoring layer for report extraction and comparison	False positives, workflow safety, automation bias, technical-context variability	LLM-assisted auditing showed very high extraction accuracy. Aidoc identified some cases missed by radiologists, but false positives were frequent in the adjudicated AI-positive sample and varied by scanner/manufacturer and artefact burden, supporting continuous real-world surveillance.	Retrospective design; adjudication limited to sampled AI-positive cases; no full system-wide false-positive rate or long-term drift estimate
Cook et al., 2026 [47]	USA; Mayo Clinic enterprise radiology practice; 17 internally developed imaging AI tools	Live post-deployment evaluation	Descriptive implementation case series; enterprise portfolio averaging > 10,000 runs per week	Live deployment within enterprise radiology infrastructure with dashboards, alerts, service tickets, and human review	Technical failure, algorithmic failure, data drift, workflow harm, end-user feedback, governance review	Structured surveillance detected routing failures, architecture-related breakdowns, scanner/protocol drift, and workflow harm from failed outputs. In one example, failed AI segmentation increased median turnaround time from 19 min to 44 min, illustrating that technical failure can translate directly into operational harm.	Single-institution descriptive experience; limited direct patient-outcome or diagnostic-accuracy evaluation
Thomas et al., 2023 [46]	UK; two NHS hospitals; AIaMD for dermoscopic skin lesion assessment and triage	Live post-deployment evaluation	Prospective service evaluation/post-market surveillance study; 14,500 cases overall; 8571 lesions with confirmed outcomes	DERM deployed live in urgent skin-cancer pathways with second-read safety net and repeat-presentation surveillance	Diagnostic performance, discharge safety, post-market surveillance, equity concerns, monitoring after updates	DERM showed very high sensitivity for melanoma and malignancy and enabled discharge of a subset of benign lesions, reducing specialist workload. No DERM-discharged lesions later re-presented as cancer during the surveillance period. The study is especially valuable for outlining a practical post-market surveillance framework with second-read safeguards, repeat-presentation searches, and active false-negative review. Equity concerns included limited evidence for darker skin types and exclusion of palmoplantar and subungual lesions.	Manufacturer involvement; non-randomised design; incomplete baseline operational comparators; residual selection and verification bias
Schreier et al., 2025 [45]	USA; community hospital; AI-powered EHR search and summarisation	Live post-deployment evaluation	Mixed-methods implementation case study over 5 months; 176 clinicians granted access	Tool embedded in live EHR pilot; evaluation emphasised adoption, satisfaction, and workflow fit	Adoption, usability, perceived time saving, onboarding, training needs	Uptake was rapid and user experience improved substantially after deployment, showing that post-deployment evaluation should include usability, workflow fit, and training needs, not technical performance alone.	Single-centre case study focused on user experience rather than clinical effectiveness, safety events, calibration, fairness, or performance drift; several authors had commercial affiliations with Google and MEDITECH
Schinkel et al., 2023 [48]	Netherlands; academic emergency departments; ML tool for blood-culture stewardship	Implemented shadow-mode post-deployment monitoring	Prospective real-time longitudinal monitoring study; 3035 ED visits over 1 year	Model generated predictions in real time within the EHR, but outputs were hidden from clinicians and monitored via dashboard	Temporal performance stability, case-mix change, outcome-rate change, need for recalibration	Discrimination and calibration remained stable over one year despite changing practice and case mix. The study supports statistical process control as a practical surveillance method for implemented clinical ML tools running in shadow mode.	Single-centre study; one-year follow-up; shadow-mode rather than clinician-facing use; no direct patient-outcome assessment
Kwong et al., 2022 [19]	Canada; paediatric urology; deep-learning ultrasound model for obstructive hydronephrosis	Silent or shadow-mode prospective evaluation	Prospective silent-trial study with iterative refinement; 523 kidneys in Silent Trial 1 and 711 in Silent Trial 2	Predictions generated prospectively but concealed from clinicians before any live clinical use	Generalisability failure, dataset drift, subgroup bias, feasibility, stakeholder attitudes	A model that performed well retrospectively failed severely in the first silent trial, then improved after preprocessing/model revision. The study provides a strong example of why silent prospective evaluation can identify hidden risk before patients are exposed to model-guided care.	Single-centre design; outcome partly linked to surgical decision-making; patient questionnaire unvalidated
Chi et al., 2022 [51]	USA registry oncology data; ML survival prediction models across four cancers	Retrospective temporal monitoring/updating	Longitudinal methodological study across 16 temporal cohorts; four large SEER cancer datasets	Not a live deployment; simulated maintenance and updating across successive future cohorts	Calibration drift, temporal deterioration, updating strategy performance, data-distribution change	Discrimination remained fairly stable but calibration deteriorated over time. Lifelong machine learning updating generally outperformed comparator strategies, supporting continuous monitoring of both performance and underlying data distribution.	Retrospective temporal evaluation; no prospective live implementation; updating still lagged behind drift occurrence
Kim et al., 2025 [49]	USA; methodological simulation study of binary clinical prediction models linked to treatment recommendations	Simulated post-deployment monitoring	Repeated simulation experiments; 1000 runs with synthetic datasets	Not a live clinical deployment; the study simulated silent and loud deployment, feedback loops, and retraining workflows	Feedback-loop bias in surveillance, performance misestimation, retraining risk	Standard monitoring markedly misestimated post-deployment performance under feedback loops and could trigger harmful retraining. Feedback-aware strategies recovered performance more accurately and better supported retraining decisions.	Simulation-based rather than real-world deployed data
Kore et al., 2024 [40]	Canada; chest radiography AI for pathology classification	Retrospective empirical monitoring study	Longitudinal imaging dataset of 239,235 chest radiographs plus synthetic drift experiments	Evaluated surveillance methods intended for deployed imaging AI, but not a live interventional deployment	Input drift detection, temporal shift, sensitivity of monitoring methods, value beyond aggregate performance	Aggregate performance metrics alone were poor proxies for drift. Combined image- and output-based methods were generally more sensitive, supporting explicit drift surveillance when reliable labels are delayed or unavailable.	Restricted to chest radiograph computer vision; limited demographic exploration; no direct patient-impact assessment
Subasri et al., 2025 [11]	Canada; seven hospitals; dynamic deep-learning mortality prediction from longitudinal EHR data	Simulated deployment with prospective rolling-window updating	Retrospective methodological study; 143,049 adult inpatients	Simulated surveillance and updating framework rather than live clinician-facing deployment	Harmful data-shift detection, subgroup deterioration, transfer learning, continual learning, fairness	Clinically meaningful shifts could be detected before dependence on delayed labels, and drift-triggered updating outperformed fixed-interval updating and locked-model strategies. The study is notable for linking shift detection to actionable remediation.	Simulated deployment rather than live implementation; updating thresholds may not generalise; risk of overfitting, forgetting, and feedback effects remains
Zech et al., 2018 [44]	USA; multicentre chest radiograph CNN for pneumonia detection	Pre-deployment robustness/safety evaluation	Retrospective multi-institution external validation study; 158,323 radiographs	Not live deployment; designed to assess cross-site generalisability before clinical use	External validity, confounding, spurious feature learning, calibration across settings	Internal performance overstated external performance, and models could exploit hospital-specific signals rather than pathology. The study remains a key warning that apparently high-performing models may fail unsafely across institutions.	Retrospective study; stronger on pre-deployment robustness risk than on real-world deployment or monitoring
Oakden-Rayner et al., 2022 [41]	Australia and USA; deep-learning detection of proximal femoral fracture on radiographs	Pre-deployment robustness/safety evaluation	Preclinical diagnostic-accuracy study with reader study, external validation, and algorithmic audit	Not live deployment; explicit safety evaluation before possible interventional use	Hidden failure modes, operating-point instability, subgroup error, algorithmic audit	Despite very high apparent discrimination, external operating-point instability and subgroup failures exposed clinically important hidden risk. The study shows that algorithmic auditing can reveal unsafe behaviour not evident from summary accuracy alone.	Preclinical design; no evidence on live workflow use, clinician interaction, or longitudinal monitoring
Wang et al., 2025 (oncologic radiology) [38]	China; multicentre evaluation of large reasoning models for oncologic report impression generation	Pre-deployment controlled evaluation	Retrospective multicentre study; 990 cases; reader assessment by six radiologists	Not live deployment; human-in-the-loop comparison of reasoning versus conclusion-only outputs	Diagnostic completeness, reasoning–conclusion mismatch, workflow burden, automation-bias risk	Reasoning-based outputs improved completeness and interpretability versus conclusion-only outputs, but clinically important residual errors remained, including cases where correct reasoning yielded a wrong final conclusion. Greater transparency therefore did not eliminate safety risk.	Retrospective design; no live implementation, longitudinal monitoring, or patient-outcome assessment
Wang et al., 2026 (brain MRI) [37]	China; multicentre evaluation of LLM-generated diagnostic impressions from brain MRI findings	Pre-deployment controlled evaluation	Retrospective benchmark study; 4293 reports; reader study of 500 reports with six radiologists	Not live deployment; simulated AI-assisted reporting	Human–AI interaction, disease-specific failure modes, time efficiency, automation-bias risk	AI assistance improved diagnostic performance and reduced reading time, particularly for junior radiologists, but non-trivial incorrect alterations remained. The study highlights potential workflow gains alongside the risk of unsafe propagation of LLM errors.	Retrospective controlled setting; no prospective live deployment or longitudinal monitoring
Salwei et al., 2025 [43]	USA; health-system algorithmovigilance platform (VAMOS) for implemented healthcare AI	Governance and monitoring infrastructure	Human-centred design and prototype-development study involving planning sessions, stakeholder interviews, and heuristic evaluation	Not an evaluation of a single model; institutional platform intended to support monitoring of deployed AI	Dashboard requirements, anomaly alerts, fairness monitoring, escalation pathways, governance workflows	Stakeholders prioritised monitoring systems that move beyond technical accuracy to include process, outcomes, fairness, feedback capture, and maintenance logging. The study offers a concrete operational blueprint for algorithmovigilance infrastructure.	Prototype/design study; platform not yet evaluated in routine real-world use
Ramsay et al., 2025 [42]	England; NHS chest diagnostic AI rollout across imaging networks and Trusts	Early implementation and evaluation-readiness study	Rapid mixed-methods implementation evaluation; 10 networks; six Trust-level in-depth case sites	Real-world deployment was partial and often delayed; some sites used shadow mode before going live	Procurement, governance, integration, training, patient communication, evaluation readiness, organisational capacity	Procurement and rollout were slower and more resource-intensive than anticipated. The study shows that safe diagnostic AI deployment depends as much on infrastructure, governance, training, and data readiness as on model performance.	Early-phase evaluation; limited ability to assess sustained use, patient outcomes, costs, or long-term drift
Kim et al., 2025 [39]	Canada; health-system governance for clinical and operational AI solutions	Organisational governance development	Action case research/qualitative implementation study with interviews and co-design workshops	Focused on governance for several vendor AI products already in use rather than one technical model	Lifecycle oversight, monitoring processes, decommissioning, ethics, equity, role clarity	The organisation had multiple AI products in use but lacked standardised governance and monitoring. The study shows how a health system can formalise oversight structures, policies, and lifecycle responsibilities across adoption, monitoring, updating, and decommissioning.	Organisational case study; limited direct evidence on model performance, drift, or patient outcomes
Hwang et al., 2026 [36]	USA; national hospital-level AI implementation landscape	System-level readiness and monitoring-capacity study	Cross-sectional geospatial implementation study with exploratory longitudinal quality-trend analysis; 3560 hospitals	Not model-specific; assessed institutional use of predictive AI, evaluation practices, and post-implementation monitoring	Adoption disparities, infrastructure readiness, evaluation gaps, accountability structures, equity implications	AI implementation was geographically uneven and less common in higher-need communities. Interoperability was the strongest predictor of adoption, while many hospitals reported no or uncertain evaluation of model accuracy, bias, or post-implementation monitoring, indicating substantial readiness and oversight gaps.	Self-reported survey data; not model-specific; cannot determine whether implemented AI was well validated, integrated, or outcome-improving
**Abbreviations:** AI = artificial intelligence. AIaMD = artificial intelligence as a medical device. EHR = electronic health record. LLM = large language model. NHS = National Health Service.							
