# Supplementary material for: Clinical AI Beyond Development: A Scoping Review of Deployment-Related Robustness, Algorithmovigilance, and Lifecycle Oversight
Source: Healthcare (Basel). 2026 Jul 8;14(14):2052. doi: 10.3390/healthcare14142052 (PMC13409740; doi:10.3390/healthcare14142052)
Supplement: Supplementary file 1 [file healthcare-14-02052-s001.zip › healthcare-4326906-supplementary Tables-main.pdf]

Supplementary Table S1. Data-charting form used for extraction

Data-charting domain	Extracted item
Bibliographic information	Author; year of publication; article title; journal
Geographic and clinical setting	Country; healthcare setting; clinical specialty or application area
AI system characteristics	AI modality; model type; clinical task; intended use
Study design and scale	Study design; sample size; dataset size; population or data source
Deployment phase	Development-stage, external validation, bridge/silent/shadow mode, live deployment, post-deployment monitoring, simulated monitoring, or governance/implementation context
Evidence stratum	Direct live or post-deployment monitoring; near-live bridge study; methodological monitoring and maintenance; deployment-relevant robustness/predeployment safety; governance, implementation, readiness, and human-factors evidence
Workflow context	Whether the AI system was embedded in clinical workflow; whether outputs were visible to clinicians; whether outputs influenced care
Monitoring or robustness focus	Performance monitoring; calibration; drift; updating; safety; workflow; usability; fairness; governance; corrective response
Performance and safety indicators	Discrimination; calibration; threshold stability; sensitivity/specificity; false positives/false negatives; safety events; diagnostic errors

	Deployment-related robustness dimensions		Transportability; subgroup stability; temporal resilience; operational reliability; technical reliability; human–AI interaction; sociotechnical safety
	Failure or instability type		Data drift; concept drift; calibration decay; subgroup failure; workflow mismatch; automation bias; technical failure; reasoning–conclusion misalignment; incorrect AI-generated output
	Governance and corrective pathways		Monitoring ownership; escalation route; review process; recalibration; retraining; route modification; human oversight; decommissioning or updating process
	Principal findings		Main findings relevant to deployment-related robustness, monitoring, algorithmovigilance, or lifecycle oversight
	Key limitations		Study limitations reported by authors and limitations relevant to inference strength
	Relevance to review objectives		Relevance to Objective 1: risks, unintended consequences, and robustness challenges; relevance to Objective 2: monitoring, updating, auditing, governance, or lifecycle oversight
	Notes for synthesis		Codes, descriptive themes, analytical theme contribution, and evidence-stratum interpretation

Supplementary Table S2. Search log and record counts

Source	Platform	Date searched	Lower date limit	Upper date limit	Main filters applied	Records exported or identified
**MEDLINE**	PubMed	28 February 2026	No lower date restriction within PubMed/MEDLINE coverage	28 February 2026	English; AI, clinical, deployment, monitoring, drift, governance, and oversight terms	238
**Embase**	Elsevier Embase	28 February 2026	No lower date restriction within Embase coverage	28 February 2026	English; humans; AI, clinical, deployment, monitoring, drift, governance, and oversight terms	333

**Scopus**	Elsevier Scopus	28 February 2026	No lower date restriction within Scopus coverage	28 February 2026	English; title/abstract/keyword fields; AI, clinical, deployment, monitoring, drift, governance, and oversight terms	395
**Web of Science Core Collection**	Clarivate Web of Science	28 February 2026	No lower date restriction within Web of Science Core Collection coverage	28 February 2026	English; article/early access; topic search fields; AI, clinical, deployment, monitoring, drift, governance, and oversight terms	248
**Database-search total before deduplication**	—	—	—	—	—	1214
**Additional records identified through other sources**	Reference lists; Scopus; Web of Science Core Collection	28 February 2026	Not applicable	28 February 2026	Backward reference-list screening of included studies and key related reviews; forward citation tracking in Scopus and Web of Science Core Collection	0
**Duplicates removed**	Rayyan-assisted and manual verification	—	—	—	DOI, title, author, year, journal, PMID, and publication-status checks	586
**Records after duplicates removed**	Rayyan screening library	—	—	—	Deduplicated records available for screening	628
**Records screened**	Rayyan screening library	—	—	—	Title-and-abstract screening	628
**Records excluded at title-and-abstract screening**	Rayyan screening library	—	—	—	Not relevant to review objectives; model development only; internal validation only; non-clinical AI; commentary/review/protocol; no empirical deployment relevance	475
**Full-text articles assessed for eligibility**	Full-text review	—	—	—	Full-text eligibility assessment using prespecified criteria	153
**Full-text articles excluded**	Full-text review	—	—	—	Not original empirical study/no original data: 36; pure model development or internal validation only: 34; not substantively relevant to post-development evaluation or lifecycle oversight: 25; no clear deployment or translational relevance: 18; non-clinical or administrative-only AI context: 12; non-English or inaccessible full text: 10	135
**Studies included in synthesis**	Final included evidence base	—	—	—	Empirical studies and empirically grounded implementation or monitoring reports meeting eligibility criteria	18

Note: The database-search total of 1214 records represents the records exported from MEDLINE, Embase, Scopus, and Web of Science Core Collection before deduplication. Backward reference-list screening and forward citation tracking were used as supplementary search methods; however, no additional records were identified through other sources. After duplicate removal, 628 records were screened, 153 full-text articles were assessed for eligibility, and 18 studies were included in the final synthesis. Search results may differ if the same strategies are rerun later because bibliographic databases are continuously updated and may change indexing status, early-access records, document-type classification, and interface behaviour.
